# Supplementary material for: About the Accuracy and Problems of Consumer Devices in the Assessment of Sleep
Source: Sensors (Basel). 2019 Sep 25;19(19):4160. doi: 10.3390/s19194160 (PMC6806072; doi:10.3390/s19194160)
Supplement: Supplementary file 1 [file sensors-19-04160-s001.pdf]

Supplementary Material

About the accuracy and problems of consumer devices in the assessment of sleep

Mohamed S. Ameen[1], Lok Man Cheung[1,2], Theresa Hauser [1], Michael A. Hahn[1], Manuel Schabus[1, 3,]*

[1] University of Salzburg, Department of Psychology, Laboratory for Sleep, Cognition and Consciousness Research, Hellbrunner Strasse 34, 5020 Salzburg.

[2] School of Psychology, University of Surrey, Surrey, United Kingdom, Stag Hill, Guildford GU2 7XH, UK.

[3] Center for Cognitive Neuroscience Salzburg (CCNS), Hellbrunner Strasse 34, 5020 Salzburg.

* Correspondence: manuel.schabus@sbg.ac.at; Tel: (+43) 66280445113

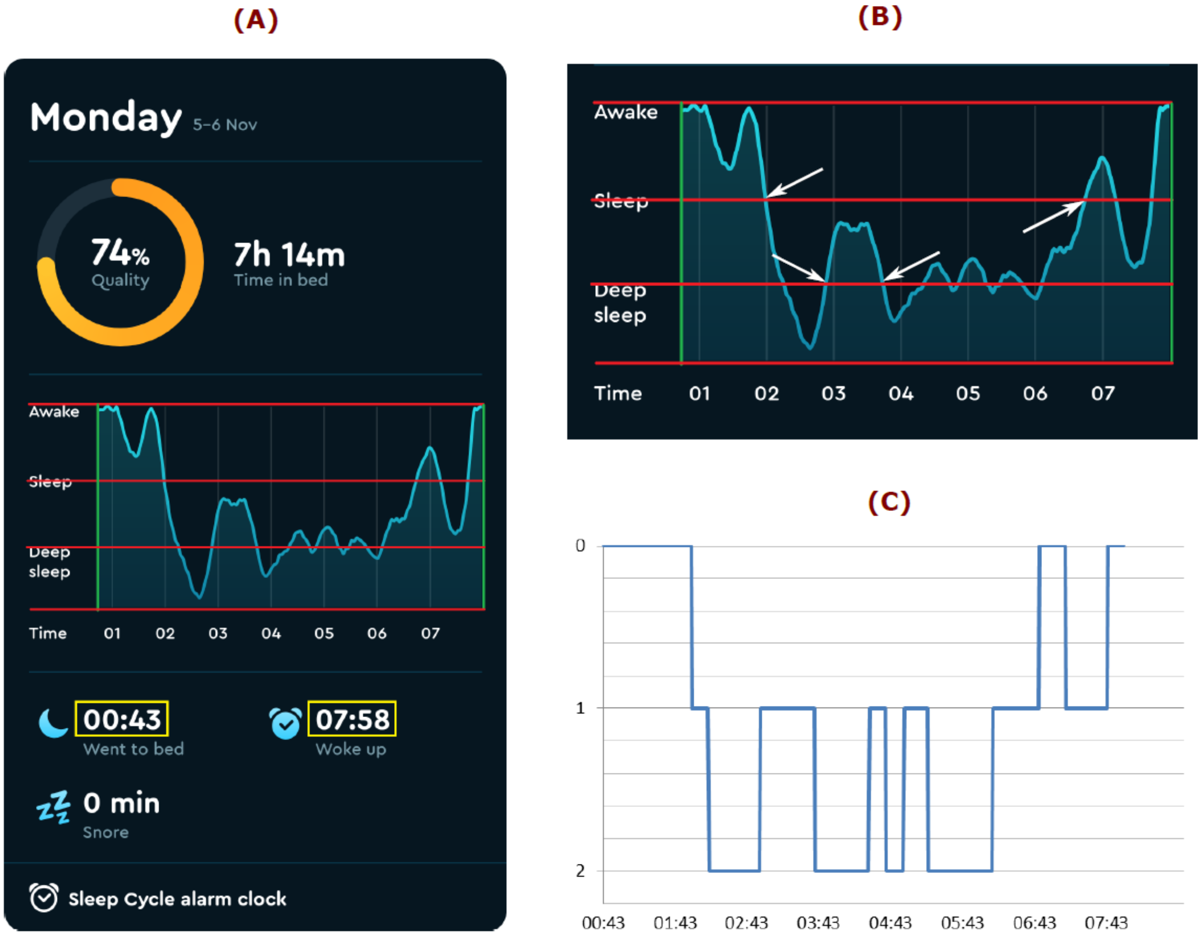

Figure S1. Sleep scoring procedure of the sleep cycle application output hypnogram. A) First we defined the boundaries of the generated hypnogram, i.e. Left, right, upper and lower boundaries, using the pixel-wise coordinates. The start and end time of the hypnogram (green lines) were defined as the time the participants went to bed and the time when they woke up respectively as reported by the application (yellow boxes). These times were then used to define the boundaries of the 30s epochs. More specifically, we calculated the overall duration of the hypnogram in minutes (time from going to bed till waking up), then calculated the total number of pixels between the left and the right boundaries of the hypnogram. Then we determine the number of pixels in 30s and these numbers were used to divide the hypnogram into 30s epochs. Furthermore, the upper and lower boundaries of the hypnogram were used to define the limits of the different sleep stages such that the distance between the upper and lower boundaries is divided by the number of stages to give the same area for each sleep stage in pixels. B) A switch from one stage to the other is detected when the tracker in the hypnogram crosses the boundary between two stages (white arrows as examples) to generate the classical 30s-epoch hypnogram depicted in

(C). This analysis was performed using image processing tools in Microsoft Paint software (Microsoft Office Professional Plus 2010) and the Visual Basic for Applications (VBA) software in Microsoft Excel (Microsoft Office Professional Plus 2010).

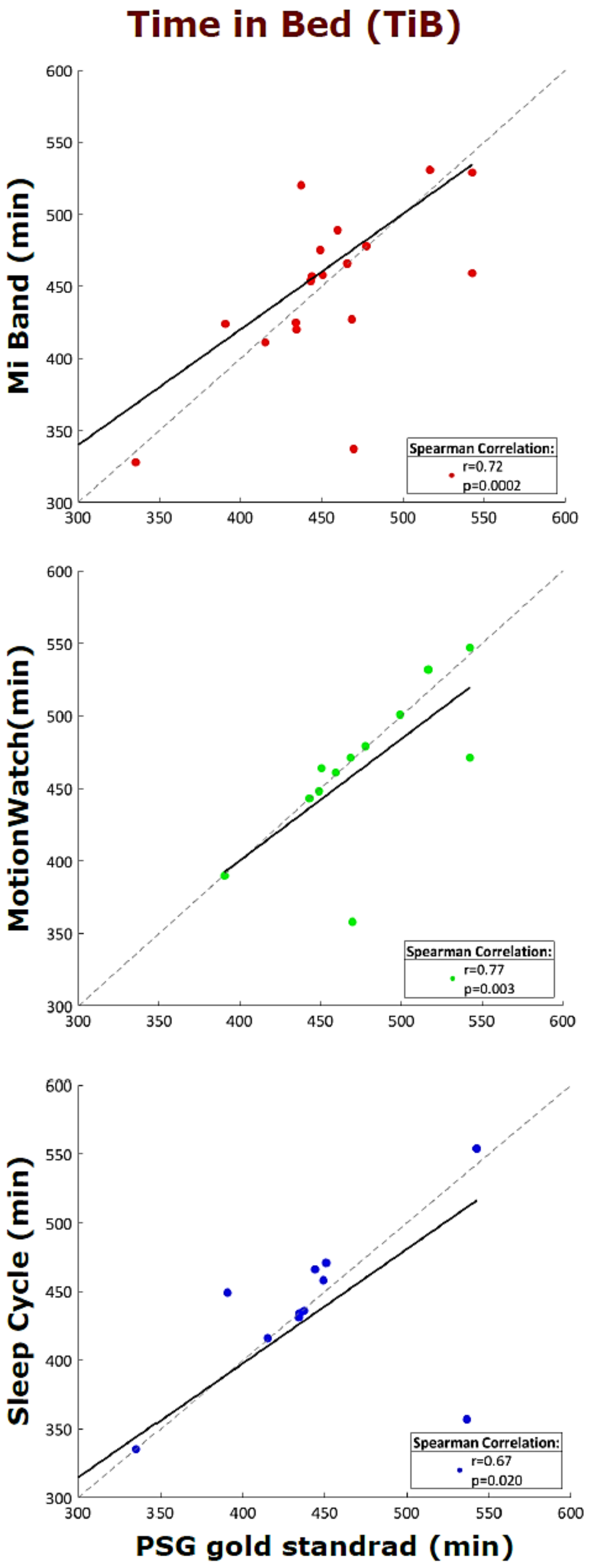

Figure S2. Scatter plot showing the positive correlations between the time in bed measurement of the PSG gold standard and those of the Mi Band, MotionWatch and sleep cycle (top to bottom). The solid black line depicts the line of correlation between the two measurements while the grey dashed line depicts the 45° line of identity.

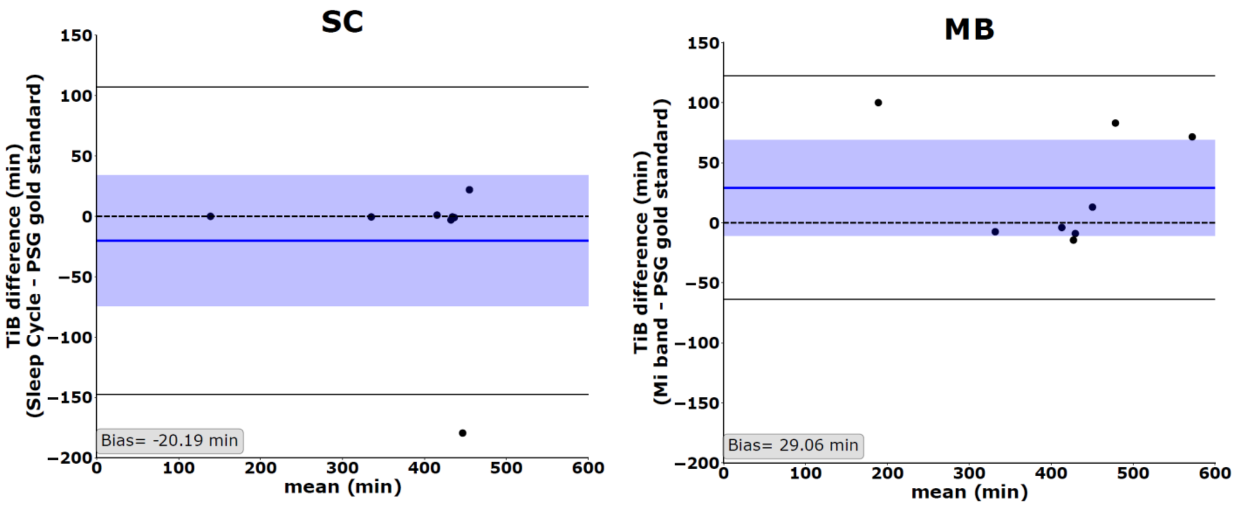

Figure S3. Bland and Altman plots for the agreement between the time in bed (TiB) as measured by the Sleep Cycle application (left) and the mi band (right) only for the at-home sleep recordings (n=8). Dashed line represent line of equality (difference = 0). Blue line is the mean of the difference and the blue shading is the confidence interval of the mean. Black horizontal lines mark the 1.96SD from the mean.

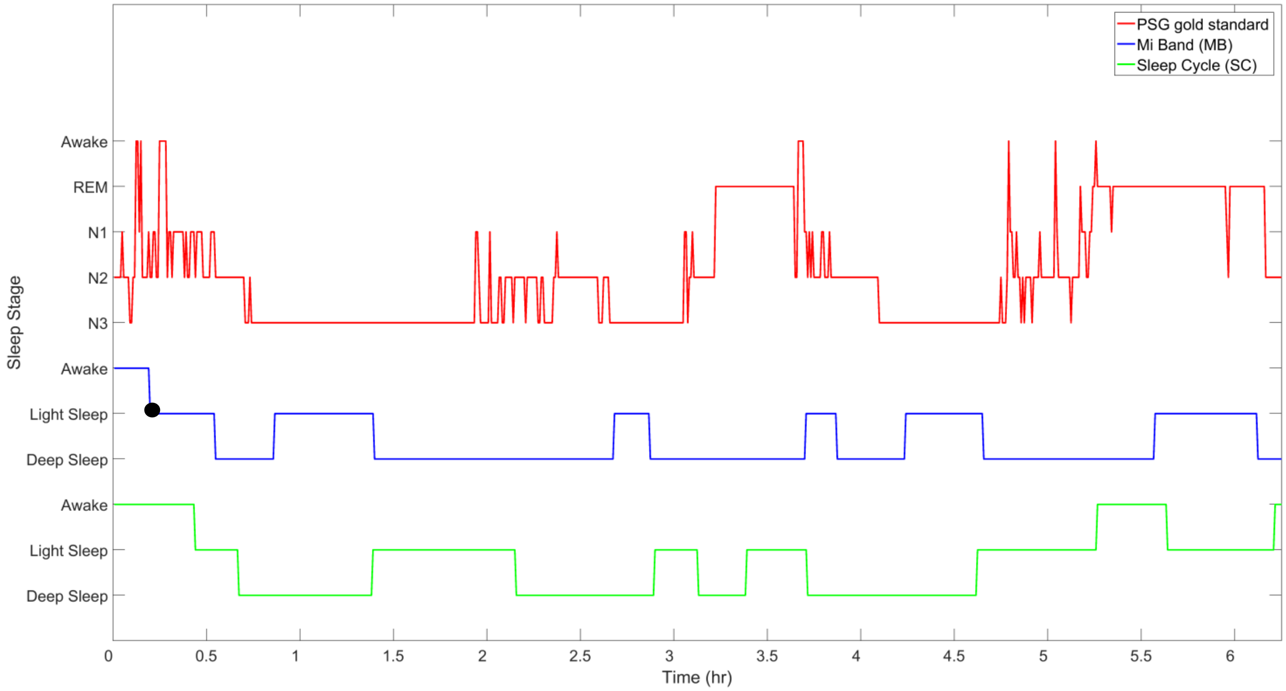

Figure S4. Hypnograms produced by the PSG gold standard (red), the Mi band (MB, blue) and the Sleep cycle application (SC, green), for the same night. An example of an unusual night with bad agreement of the PSG scores with the MB and the SC scores. Note the inability of the MB and the SC to detect the transient changes in the sleep architecture throughout the night. Moreover, The MB and the SC were inaccurate in capturing wake after sleep onset (WASO) and the sleep onset latency (SOL), the reason why they tend to over-/under-estimate such parameters (Figure 3). Also note that the MB started staging sleep with a light-sleep epoch 23 minutes after the PSG started recording (the black dot), which means that the previous time the MB was not able to detect any sleep and did not perform any sleep scoring, another problem that might contribute to the poor agreement of the MB scoring with the gold standard. Here, we assumed this time to be “awake” for clarification.

Table S1. Spearman correlation results between the PSG gold standard and the Mi Band, the MotionWatch and the Sleep Cycle application for the key sleep parameters.

**Parameter**	**Mi Band**	**MotionWatch**	**Sleep Cycle**
**SOL**	r_{19} = -0.06<br>p = 0.78	r_{10} =-0.09<br>p = 0.76	r_{10} =0.34<br>p= 0.27
**WASO**	r_{19} = 0.28<br>p = 0.22	**r_{10} = 0.78**<br>**p = 0.02**	r_{10} =0.41<br>p = 0.17

**SE**	$r_{19} = 0.43$<br>$p = 0.052$	$r_{10} = 0.33$<br>$p = 0.28$	$r_{10} = 0.32$<br>$p = 0.29$
**TST**	$\mathbf{r_{19} = 0.49}$<br>$\mathbf{p = 0.02}$	$r_{10} = 0.41$<br>$p = 0.17$	$r_{10} = 0.27$<br>$p = 0.39$
**TiB**	$\mathbf{r_{19} = 0.72}$<br>$\mathbf{p = 0.0002}$	$\mathbf{r_{10} = 0.77}$<br>$\mathbf{p = 0.03}$	$\mathbf{r_{10} = 0.67}$<br>$\mathbf{p = 0.02}$

SOL: Sleep onset latency, WASO: wake after sleep onset, SE: sleep efficiency and TST: total sleep time. TiB: time in bed. In bold are the significant correlations.

Table S2. Epoch-wise agreement Sleep/wake while discarding epochs scored as REM by the PSG gold standard. Note the difference in the OA and K scores as compared to Table 2 in the main article.

	**PSG gold standard**	
	**WAKE**	**SLEEP**
**Mi Band (MB) staging**		
**Wake**		
**% Sensitivity**	**5.5**	**0.6**
**% PPV**	**62.8**	**37.2**
**Sleep**		
**% Sensitivity**	**94.5**	**99.4**
**% PPV**	**15.2**	**84.8**
**Sleep Cycle (SC) staging**		
**Wake**		
**% Sensitivity**	**55.6**	**30.1**
**% PPV**	**24.3**	**75.7**
**Sleep**		
**% Sensitivity**	**44.4**	**69.9**
**% PPV**	**9.9**	**90.1**

**MotionWatch (MW) staging**		
**Wake**		
**% Sensitivity**	**35.7**	**8.7**
**% PPV**	**43.8**	**56.2**
**Sleep**		
**% Sensitivity**	**64.3**	**91.3**
**% PPV**	**11.8**	**88.2**

**Device/Application**	**OA (%)**	**K (PABAK)**
**Mi Band** **MB**	**84.54**	**0.08 (0.68)**
**Sleep Cycle** **SC**	**67.79**	**0.17 (0.34)**
**MotionWatch** **MW**	**81.92**	**0.32 (0.62)**

Table S3. Epoch-wise agreement comparison between (Wake/light Sleep/Deep sleep) and (Sleep/wake).

	**PSG gold standard**			
	**WAKE**	**LIGHT SLEEP**	**DEEP SLEEP**	**SLEEP**
**MiBand (MB) staging**				
**Wake**				
**% Sensitivity**	**5.5**	**0.1**	**1.5**	**0.6**
**% PPV**	**62.8**	**4.7**	**32.6**	**37.2**
**Light sleep (Sleep)**				
**% Sensitivity**	**79.2**	**70.6**	**51.3**	**99.4**
**% PPV**	**18.9**	**57.8**	**23.2**	**84.8**
**Deep Sleep**				
**% Sensitivity**	**15.3**	**29.3**	**47.2**	
**% PPV**	**7.5**	**48.9**	**43.6**	

Sleep Cycle (SC) staging

**Wake**				
% Sensitivity	55.6	37.0	16.9	30.1
% PPV	24.3	61.1	14.7	75.7
**Light sleep (Sleep)**				
% Sensitivity	36.4	40.9	31.1	69.9
% PPV	14.4	61.2	24.4	91
**Deep Sleep**				
% Sensitivity	8.0	22.1	52.0	
% PPV	4.1	42.8	53.0	

MotionWatch (MW) staging

**Wake**				
% Sensitivity	35.7	--	--	8.7
% PPV	43.8	--	--	56.2
**Sleep**				
% Sensitivity	64.3	--	--	91.3
% PPV	11.8	--	--	88.2

Device/Application	(W/LS/DS) OA (%) / K (PABAK)	(W/S) OA (%) / K (PABAK)
mi-band MB	53.02 / 0.17 (0.06)	86.54 / 0.08 (0.72)
Sleep Cycle SC	46.34 / 0.18 (-0.08)	65.90 / 0.13 (0.30)
MotionWatch MW	--	83.42 / 0.33 (0.66)

We pooled Light and Deep sleep into one category labelled 'Sleep' and measured the agreement when the devices are only required to assign each epoch to one of two categories (Wake/Sleep) instead of 3 categories (Wake/Light sleep/Deep sleep).

Table S4. A comparison of sleep architecture between Laboratory and home PSG.

Parameter	Laboratory PSG (n=17)	Home PSG (n=8)	P*
**TiB**	452.29 ± 81.78 min	396.94 ± 117.54 min	0.26
**TST**	378.5 ± 95.21 min	352.31 ± 127 min	0.61
**SOL**	30 ± 20.49 min	17.44 ± 14.19 min	0.09⁺
**WASO**	44.44 ± 38.66 min	27.69 ± 37.80 min	0.32
**SE**	82.41 ± 13.83%	87.63 ± 11.89%	0.35

*Independent sample t-test without assuming equal variances (Welch approximation t-test). ⁺p-value < 0.1 showing a statistical trend. SOL: Sleep onset latency, WASO: wake after sleep onset, SE: sleep efficiency and TST: total sleep time. TiB: time in bed.

Table S5. A comparison between sleep parameters as measured by the PSG gold standard and the 3 sleep trackers; Mi band, MotionWatch and Sleep cycle.

Parameter	PSG (n=25)	MB (n=21)	SC (n=12)	MW (n=12)
**TiB (min)**	434.58 ±13.22	456.38 ±89.79	412.17 ±102.23	472.10 ±41.16
**TST(min)**	370.12 ±104.43	447.28 ±87.11	260.75 ±101.10	417.17 ±35.81
**SOL (min)**	25.98 ±19.35	11.24 ±20.18	30 ±18.70	17.10 ±20.73
**WASO (min)**	39.10 ±38.42	9.14 ±20.24	121.33 ±44.94	43.75 ±24.86

**SE (%)**	84.10 ±13.22	97.92 ±4.53	60.29 ±15.32	88.47 ±5.19

SOL: Sleep onset latency, WASO: wake after sleep onset, SE: sleep efficiency and TST: total sleep time, TiB: time in bed, PSG: polysomnography, MB: Mi Band, SC: Sleep Cycle, MW: MotionWatch.

Table S6. The amount of time slept by each participant before and after removing the time spent in REM "dreaming" sleep.

**TST with REM**	**TST without REM**
383.5	317
81.5	81.5
432.5	373.5
423	375.5
335.5	305.5
502	438
438	386.5
381.5	302
307.5	259.5
504	441.5
337	301.5
420.5	389
317.5	266
386	307.5

391.5	329
362	303.5
431	353.5
425	316.5
390	295
419.5	364.5
508.5	455
205.5	190
398	331.5
120.5	112
351.5	310

Table S7. A table showing the agreement between the sleep scoring of a manual scorer and the scoring of the sleep trackers in classifying 30s epochs into 3 categories (Wake/light sleep/deep sleep).

	Manual scorer		
	**WAKE**	**Light Sleep**	**Deep Sleep**
**Mi Band (MB) staging**			
**Wake**			
**% Sensitivity**	**5.3**	**0.2**	**1.6**
**% PPV**	**59.5**	**7**	**33.5**
**Light Sleep**			
**% Sensitivity**	**85.8**	**69.5**	**49.2**
**% PPV**	**20.8**	**56.7**	**22.5**
**Deep Sleep**			
**% Sensitivity**	**9**	**30.3**	**49.3**
**% PPV**	**4.4**	**50.1**	**45.5**

**Sleep Cycle (SC) staging**			
**Wake**			
**% Sensitivity**	**59.7**	**35**	**17.4**
**% PPV**	**27.1**	**57.5**	**15.5**
**Light Sleep**			
**% Sensitivity**	**31.7**	**42.5**	**30.7**
**% PPV**	**12.9**	**62.6**	**24.5**
**Deep Sleep**			
**% Sensitivity**	**8.6**	**22.5**	**51.9**
**% PPV**	**4.5**	**42.5**	**53**

Devices/applications	OA (%)	K/PABAK
**MB**	**53.11**	**0.14/0.06**
**SC**	**47.90**	**0.21/-0.06**

Table S8. The agreement between the sleep scoring done by a manual scorer and the sleep trackers when classifying 30s epochs into two categories (Sleep/wake)

	Manual Scorer	
	**WAKE**	**SLEEP**
**Mi Band (MB) staging**		
**Wake**		
**% Sensitivity**	**5.3**	**0.7**
**% PPV**	**59.5**	**40.5**
**Sleep**		
**% Sensitivity**	**94.7**	**99.3**
**% PPV**	**15.4**	**84.6**

**Sleep Cycle (SC) staging**		
**Wake**		
% Sensitivity	59.7	28.8
% PPV	27.1	72.9
**Sleep**		
% Sensitivity	40.3	71.2
% PPV	9.2	90.8
**MotionWatch (MW) staging**		
**Wake**		
% Sensitivity	36.4	8
% PPV	53.2	46.8
**Sleep**		
% Sensitivity	63.6	92
% PPV	14.6	85.4

Device/Application	OA (%)	K/PABAK
Mi Band MB	84.26	0.07/0.68
Sleep Cycle SC	69.41	0.21/0.38
MotionWatch MW	80.98	0.32/0.61
